# A Case of Multiple Intracranial Hemorrhages Due to Biopsy-Proven Varicella Zoster Virus Vasculopathy Without Rash

**DOI:** 10.7759/cureus.77890

**Published:** 2025-01-23

**Authors:** Yumi Honda, Toshiya Nomura, Keiichi Nakahara, Junichi Matsuo, Ryo Shirahama, Kenji Kuroki, Mitsuharu Ueda

**Affiliations:** 1 Neurology, Kumamoto University, Kumamoto, JPN; 2 Neurology, Ariake Medical Center, Arao, JPN; 3 Neurology, Kumamoto Rosai Hospital, Yatsushiro, JPN; 4 Neurology, Sugimura Hospital, Kumamoto, JPN; 5 Neurology, Japanese Red Cross Kumamoto Hospital, Kumamoto, JPN

**Keywords:** brain biopsy, cerebrospinal fluid analysis, intracranial hemorrhage, varicella-zoster virus vasculopathy, vzv igg antibody index

## Abstract

Varicella zoster virus (VZV) vasculopathy is a rare complication of VZV infection that can lead to severe neurological manifestations, including multiple intracranial hemorrhages. We report the case of an 80-year-old male patient with a history of Parkinson’s disease and hypertension who presented with altered consciousness and motor difficulties. Imaging studies revealed recurrent cortical-subcortical hemorrhages in the right frontal lobe. Laboratory tests showed elevated cerebrospinal fluid (CSF) protein, increased CSF cell count, and a significantly elevated VZV IgG antibody index, while CSF VZV DNA was undetectable. A brain biopsy confirmed the diagnosis, showing vasculitis with VZV DNA positivity via real-time polymerase chain reaction (PCR) and immunohistochemistry. The patient was treated with intravenous acyclovir and corticosteroids, which partially improved clinical outcomes. This case highlights the importance of considering VZV vasculopathy as a differential diagnosis in unexplained intracranial hemorrhages, even in the absence of typical skin lesions. Comprehensive diagnostic evaluation, including CSF antibody testing and brain biopsy, is essential for accurate diagnosis and management.

## Introduction

Varicella zoster virus (VZV), a neurotropic virus, establishes latency in ganglion neurons following primary infection manifesting as chickenpox. Impairment of VZV-specific cell-mediated immunity triggers viral reactivation. While the reactivated virus conventionally spreads anterogradely along neural pathways to the dermis, producing herpes zoster, retrograde viral migration to the central nervous system may induce severe neurological complications in the absence of cutaneous manifestations [1]. VZV vasculopathy is a rare but serious condition characterized by inflammation and damage to cerebral blood vessels. This condition can manifest as ischemic or hemorrhagic stroke, often presenting diagnostic and therapeutic challenges [2-4].

VZV vasculopathy typically occurs in the context of herpes zoster, with a visible rash serving as a clinical clue. However, vasculopathy may develop without the hallmark cutaneous manifestations, complicating timely diagnosis [5,6]. Diagnosis relies on clinical presentation, cerebrospinal fluid (CSF) analysis, neuroimaging, and, in some cases, histopathological examination [7]. Key findings include an elevated VZV IgG antibody index in the CSF and evidence of vascular inflammation on biopsy.

Here, we report the case of an 80-year-old male patient who presented with recurrent intracranial hemorrhages and no associated rash. A comprehensive diagnostic workup, including brain biopsy and advanced molecular techniques, confirmed the diagnosis of VZV vasculopathy. Although this case highlights VZV vasculopathy without cutaneous manifestations, as a single case report, it cannot establish causality or prevalence. Future research is needed to validate these findings in larger cohorts.

## Case presentation

An 80-year-old Japanese man with a medical history of Parkinson’s disease, hypertension, hyperlipidemia, and prior appendicitis presented with altered consciousness and difficulty moving. Four days prior to the presentation, dizziness and mood disorders developed and gradually the patient became almost unresponsive to verbal stimuli and had difficulty moving, and thus was referred to our institution for further evaluation and management. Before the onset, he had early morning off symptoms and was stage 4 on the Hoehn and Yahr scale, but he also did farm work. There was no history of smoking or significant alcohol consumption. His home medications included levodopa-carbidopa (500 mg/day), rotigotine (9 mg/day), and pravastatin (10 mg/day). 

Initial physical examination revealed a body temperature of 37.3°C, blood pressure of 132/82 mmHg, and a Glasgow Coma Scale (GCS) score of E3V1M6. The neurological examination noted rigidity in both upper and lower limbs but no evidence of overt paralysis. No cranial nerve deficits or neck stiffness were identified. The patient denied any headache or earache, and no obvious rash was observed. 

**Figure 1:**
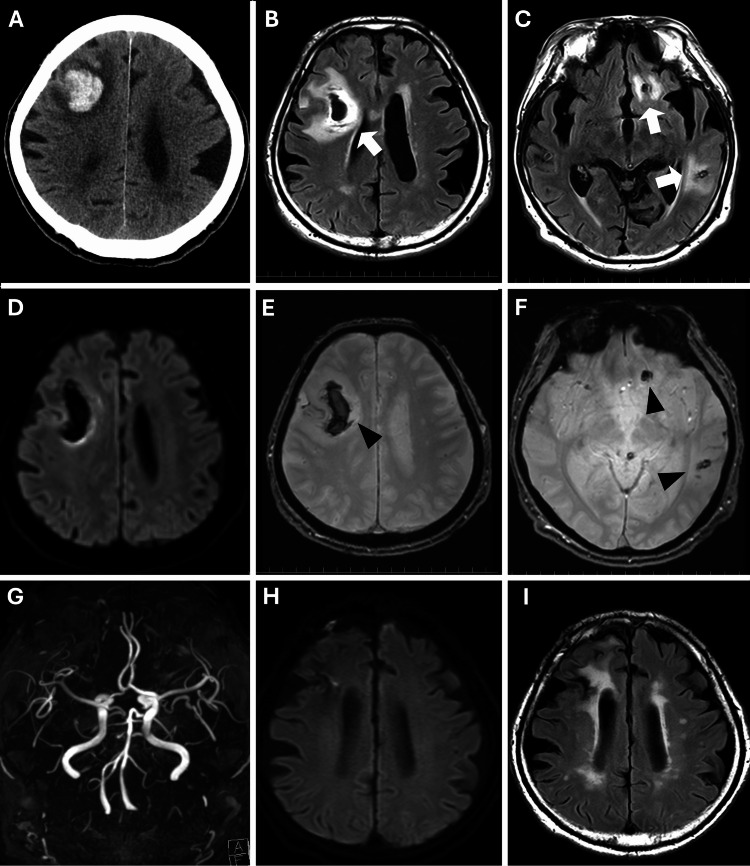
Neuroimaging findings (A) Head CT showing a large cortical-subcortical hemorrhage in the right frontal lobe. (B, C) FLAIR MRI highlighting edema and hyperintense signals surrounding the hemorrhagic lesion in the bilateral frontal lobes and the left temporal lobe (arrows). (D) DWI MRI showing low intense signal in the right frontal lobe. (E, F) T2-star MRI demonstrating susceptibility changes consistent with hemorrhages (arrowheads). (G) MRA revealing no significant abnormalities in the major cerebral arteries. (H, I) Follow-up MRI two months after treatment showed lesion reduction on both DWI and FLAIR. FLAIR: fluid-attenuated inversion recovery; DWI: diffusion-weighted imaging; MRA: magnetic resonance angiography

A head CT scan revealed a cortical-subcortical hemorrhage in the right frontal lobe with associated edema (Figure 1A). Follow-up MRI showed hyperintense signals in the same region and in the left frontal and temporal lobes on fluid-attenuated inversion recovery (FLAIR) images and lowintense signals on diffusion-weighted imaging (DWI), while T2-star images showed susceptibility-weighted changes consistent with hemorrhage (Figure 1B-1F). MRA findings indicate no significant abnormalities in the major cerebral vessels (Figure 1G). CSF analysis revealed elevated protein levels (101.9 mg/dL; reference range (RR) 10-40 mg/dL), a significant increase in cell count (46 cells/μL, 100% mononuclear; RR <5 cells/μL), an elevated IL-6 level (202 pg/mL; RR 4.3 pg/mL). However, VZV DNA was not detected in the CSF by real-time polymerase chain reaction (PCR). 

**Figure 2:**
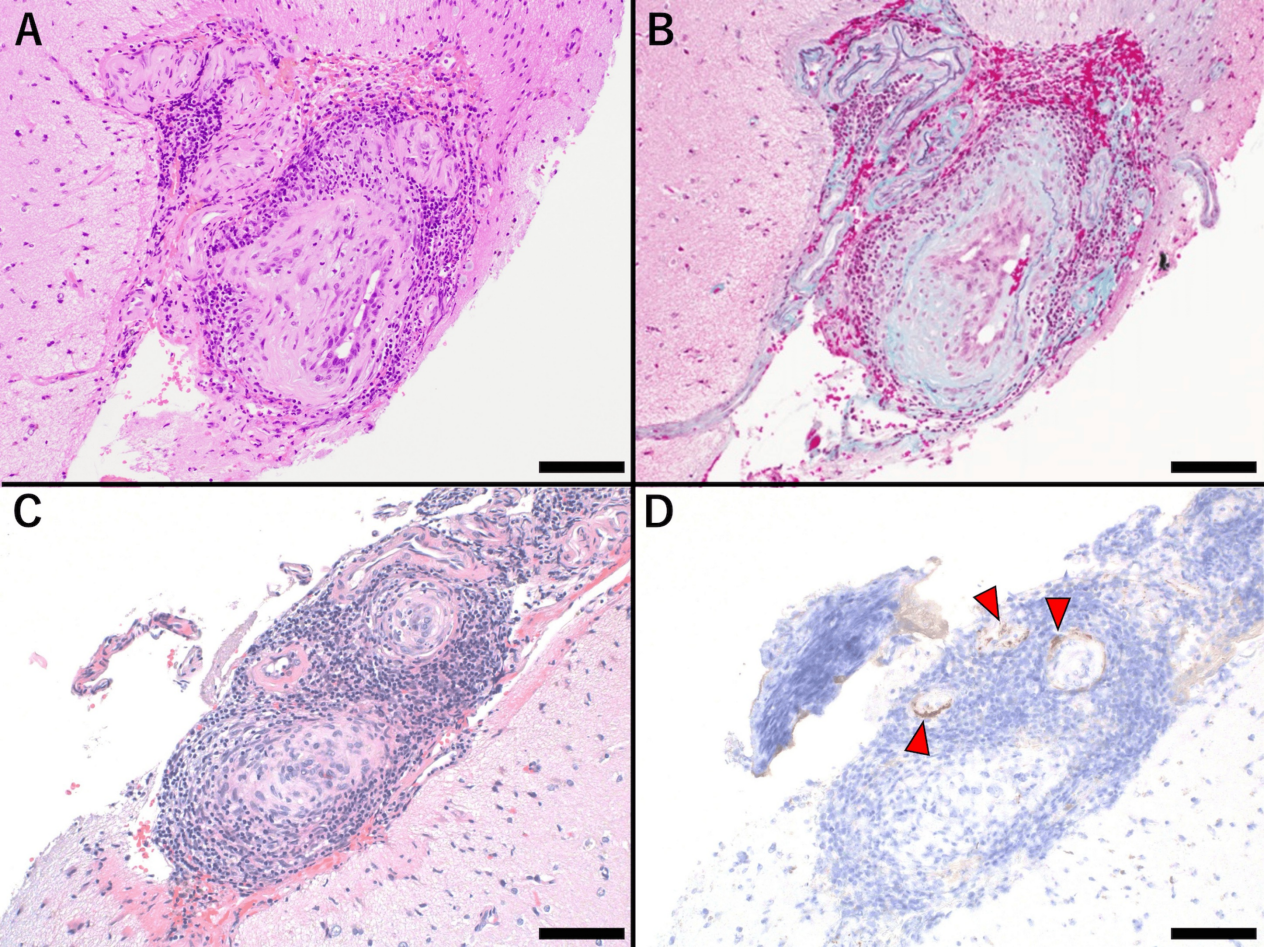
Histopathological findings of brain biopsy taken from the surface of the medial right frontal lobe confirming VZV vasculopathy. (A) H&E staining showing lymphocytic infiltration in the vessel wall with evidence of vascular inflammation. (B) EMG revealing disruption of the elastic lamina and intimal thickening, contributing to luminal narrowing. (C) Higher magnification of H&E staining showing extensive inflammatory cell infiltration around the vessel. (D) Immunohistochemical staining for VZV glycoprotein E (VZVgE) shows positive signals (arrowheads) in the vascular wall, confirming VZV infection. (scale bars = 100 µm.) VZV: varicella zoster virus; H&E: hematoxylin and eosin; EMG: elastic membrane staining

Given the initial differential diagnoses, including intravascular lymphoma and central nervous system vasculitis, a brain biopsy of the right frontal lobe lesion was undertaken to establish a definitive diagnosis. The biopsy revealed lymphocytic infiltration in the vessel walls, elastic lamina disruption, and intimal thickening, causing luminal narrowing (Figure 2A-2C). Immunohistochemistry revealed positive staining for VZV antigens in the vascular walls (Figure 2D), and real-time PCR detected VZV DNA in the brain tissue (approximately 8.59 × 10⁻² copies/cell). These findings confirmed the diagnosis of VZV vasculopathy. In both serum and CSF, VZV IgG levels exceeded the upper limit of detection, and subsequent dilutional retesting revealed a significant increase in the VZV IgG index (11.7; RR <2.0).

Three months after presentation, treatment was initiated with intravenous methylprednisolone (IVMP, 1 g/day for three days) and acyclovir (1,500 mg/day), followed by oral prednisolone (30 mg/day). While this regimen effectively prevented further cerebral hemorrhage, neurological improvement was limited. Follow-up CSF analysis after two months revealed increased protein levels (101.9 → 161.6 mg/dL; RR 10-40 mg/dL), reduced cell count (46 → 8 cells/µL; RR <5 cells/μL), and decreased IL-6 levels (202 → 2.7 pg/mL; RR 4.3 pg/mL). Follow-up MRI two months after treatment initiation demonstrated a reduction in lesion size on both DWI and FLAIR (Figure 1H, 1I). Although consciousness improved (GCS E3V1M6 → E4V3M6), functional recovery remained poor. By Day 144 post-admission, the patient was still bedridden. 

## Discussion

VZV vasculopathy is a rare yet significant neurological condition caused by the reactivation of VZV within cranial nerve ganglia, leading to viral invasion of cerebral arteries [2,6,7]. This condition can result in inflammation, vessel wall damage, and subsequent ischemic or hemorrhagic strokes. Previous cases have documented VZV vasculopathy and intracranial hemorrhage occurring without cutaneous manifestations [6,8], consistent with our case.

The diagnosis of VZV vasculopathy in this patient was established based on clinical presentation, neuroimaging, CSF analysis, and histopathological findings. While VZV DNA detection in the CSF by PCR is a key diagnostic tool, its sensitivity is limited, particularly in cases where testing occurs beyond the acute phase of infection. In contrast, the VZV IgG antibody index offers greater sensitivity in detecting intrathecal antibody synthesis [2]. In a study by Nagel et al., they reported that 93% of patients with VZV vasculitis had anti-VZV IgG antibodies in the CSF, but VZV DNA was detected in only 30% [5]. Therefore, an indicative measurement of the VZV IgG antibody index should be considered when negative PCR results [9]. Brain biopsy serves as a vital diagnostic modality for establishing a definitive diagnosis of VZV vasculopathy, especially in atypical or challenging cases. The current patient’s biopsy revealed vasculitis with lymphocytic infiltration, elastic lamina disruption, and VZV antigens within vascular walls. Notably, the disruption of the internal elastic lamina is a hallmark pathological feature of VZV vasculopathy [10]. Real-time PCR confirmed VZV DNA in brain tissue, providing definitive evidence of VZV involvement. 

VZV-induced vasculopathy arises from viral replication in the vascular adventitia and subsequent spread to the media and intima, leading to vessel wall inflammation, thrombosis, and ischemia. In some cases, such as the present one, the inflammation can weaken vessel walls, resulting in intracranial hemorrhages [4,6]. The absence of rash in the current patient suggests that viral reactivation occurred in ganglia with limited cutaneous involvement, emphasizing the need for a high index of suspicion in patients with unexplained cerebrovascular events. 

The management of VZV vasculopathy involves antiviral therapy combined with anti-inflammatory treatment. Acyclovir remains the cornerstone of antiviral therapy, with adjunctive corticosteroids used to suppress vascular inflammation [7,9]. However, the optimal dose, duration of treatment, and the need for concomitant corticosteroids have not yet been established [7]. In the present case, the patient received intravenous acyclovir and methylprednisolone, followed by oral prednisolone. Despite partial improvement in consciousness, functional impairment persisted. This observation is consistent with existing reports indicating that hemorrhagic cases generally have a worse prognosis than ischemic cases. Specifically, studies have shown that good outcomes, defined as a modified Rankin Scale score of 0-2, were achieved in 61% of ischemic cases but only 40% of hemorrhagic cases. Additionally, the mortality rate was significantly higher in hemorrhagic cases, reaching 60% compared to 22% in ischemic cases [11]. 

The poor prognosis observed in the current case aligns with previous studies indicating that hemorrhagic forms of VZV vasculopathy are associated with significant morbidity and mortality [11]. Previous reports have shown that many cases of VZV vasculopathy improve with the use of antiviral drugs [5], including some improvement in this case, suggesting that delayed diagnosis and treatment may contribute to the poor prognosis.

This case highlights the importance of considering VZV vasculopathy as a differential diagnosis in patients with unexplained intracranial hemorrhages, particularly in the elderly, even in the absence of a rash. When CSF VZV DNA is negative, clinicians should pursue antibody index testing and consider brain biopsy if clinical suspicion remains high. Furthermore, this case underscores the potential role of brain tissue PCR and immunohistochemistry in confirming the diagnosis.

## Conclusions

Diagnosis of VZV vasculopathy is particularly difficult in cases without rash. Even if CSF VZV DNA is negative by PCR, it is important to assess the VZV IgG index to detect intrathecal antibody synthesis. If the diagnosis cannot be confirmed by non-invasive testing, brain biopsy may improve diagnostic accuracy and allow early treatment. This approach allows timely intervention in patients with atypical presentations of VZV vasculopathy and may lead to better neurological outcomes. Further research is needed to optimize treatment strategies and improve outcomes in this rare but severe condition. 
